# Education Research: EEG Instruction: Findings from a Scoping Review

**DOI:** 10.1212/NE9.0000000000200255

**Published:** 2025-10-24

**Authors:** Andres Fernandez, Maryam Asoodar, Gregory Laynor, Alexis Peedin, Jeffrey B. Ratliff, Urvashi Vaid, Swati A. Karmarkar, Moushumi Sur, Vivianne van Kranen-Mastenbroek, Dimitrios Papanagnou, Marian H.J.M. Majoie, Dorene F. Balmer, Satid Thammasitboon

**Affiliations:** 1 Department of Neurology, Thomas Jefferson University, Philadelphia, PA; 2 School of Health Professions Education, Maastricht University, the Netherlands; 3 NYU Health Sciences Library, NYU Grossman School of Medicine, NY;; 4 Department of Pathology, Children's Hospital of Philadelphia, University of Pennsylvania, Philadelphia;; 5 Department of Internal Medicine, Thomas Jefferson University, Philadelphia, PA;; 6 Department of Pediatrics, Texas Children's Hospital, Baylor College of Medicine, Houston;; 7 Department of Clinical Neurophysiology, Maastricht UMC+, Maastricht University Medical Center, the Netherlands;; 8 Department of Emergency Medicine, Thomas Jefferson University; Philadelphia, PA;; 9 Department of Neurology, Academic Center of Epileptology Kempenhaeghe, Maastricht UMC+, the Netherlands; and; 10 Department of Pediatrics, Children's Hospital of Philadelphia, University of Pennsylvania.

## Abstract

**Background and Objectives:**

Although EEG interpretation is a key skill in neurology, EEG instruction is variable and its landscape remains underexplored. Thus, we conducted a scoping review to expand the literature by providing a structured mapping of the EEG instruction literature and a foundation for future research in EEG instruction.

**Methods:**

We followed a structured methodological framework for scoping reviews and report the findings in accordance with the Preferred Reporting Items for Systematic Reviews and Meta-Analyses extension for Scoping Reviews. Research questions explored theoretical frameworks, methodologies, learner populations, instructional methods, and assessment approaches. Eligibility criteria were iteratively modified to focus on studies on EEG instruction. A search strategy was developed by a systematic review librarian including the following databases: Scopus, PubMed, PsycINFO, Cumulative Index to Nursing and Allied Health Literature, CENTRAL, and Education Resources Information Center. An initial search was conducted in 2021, with a subsequent updated search to cover studies from 2022 to 2024. Screening was conducted in Covidence using dyads, with discrepancies resolved by a third reviewer. Data extraction categories were developed for the included studies.

**Results:**

Thirty-three EEG instruction studies were included in this scoping review. Most studies were published since 2014, were conducted in North America, originated in single institutions, and were published in neurology/neuroscience journals. Almost half of the studies had neurology trainee learner populations, but other specialty trainees were also represented. There was a dearth of theoretical frameworks underpinning the studies, and all studies used quantitative methodologies. Instructional approaches clustered around didactic instruction, online-based instruction, and experiential learning, with several studies using multiple modalities. Assessment approaches clustered around multiple-choice question tests, tests based on EEG samples, and standardized summative tests. Several studies used multiple assessment approaches, but most were lower level (Kirkpatrick level 1 or 2).

**Discussion:**

Our scoping review reveals a surge in publications on EEG instruction with a trend toward multimodal instructional approaches, with an assessment focus on knowledge. The findings from this scoping review highlight 3 areas for growth and improvement that future research might address: increase the rigor of research on EEG educational interventions, advance the scope of EEG instruction research, and enhance the precision of EEG instruction.

## Introduction

Interpreting EEG is a crucial skill in neurology. Teaching and assessing EEG knowledge and skills among trainees is a vital component of neurology education. As a foundational diagnostic tool, errors in EEG interpretation can lead to inappropriate treatment and potential harm to patients.^1^ Despite its importance, EEG instruction—encompassing both teaching and assessment—varies significantly across training programs.^2^ Moreover, both trainees and program directors lack confidence in trainees' ability to accurately interpret EEG by the end of training.^3^ Recent literature reviews provide valuable insights into EEG instruction but have included learners in a variety of health professions and settings. For instance, a scoping review examined critical care EEG education among participants in the intensive care settings,^4^ and a systematic review evaluated educational programs that taught participants to perform or screen EEGs.^5^ However, the broader landscape of EEG instruction, particularly for neurology trainees, remains underexplored.

To address this gap in the literature, we conducted a scoping review to describe the full landscape of EEG instruction across various contexts and populations. A comprehensive mapping of the literature is crucial for understanding the depth and breadth of existing studies and identifying areas requiring further research.^6^ A scoping review is the most suitable approach for this study because of the emerging and conceptually diverse nature of EEG instruction literature; the heterogeneity of study designs, outcomes, and learner populations; and the goal to map existing research, identify gaps, and inform future scholarly and curriculum development efforts. Attempting a systematic review at this stage would risk being premature, overly restrictive, and potentially uninformative. Our review aims to serve as a foundation for future studies, particularly those exploring critical questions regarding the adequacy and consistency of EEG training and the definition of competence in EEG interpretation across different contexts and for different learner groups.

## Methods

### Protocol and Registration

Our review protocol, titled “Teaching visual diagnosis in the health professions: a scoping review,” was registered with Best Evidence in Medical and Health Professional Education. Initially, we intended to synthesize evidence on various modalities of visual diagnosis, including electrocardiography (EKG), polysomnography, and radiographic imaging, in the initial screening stage to inform EEG instruction. However, we gained insights from the literature during our pilot search and initial screening stage that led us to iteratively narrow the focus of this scoping review to waveform studies in medical education and ultimately to focus only on EEG instruction studies (details of the multistage screening process in the Figure).

**Figure:**
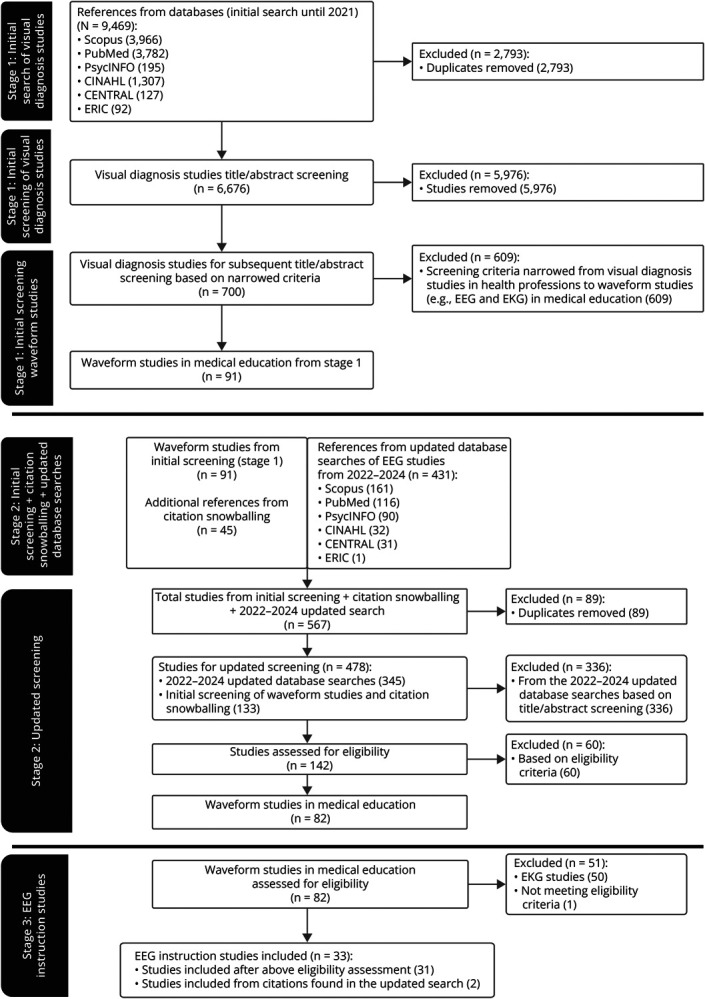
PRISMA Flow Diagram of Study Inclusion This figure describes the iterative multistage screening process leading to the 33 EEG instruction studies in this scoping review: *Stage 1 (initial screening): The top of the flowchart outlines the initial screening stage of studies of visual diagnosis in health professions education (initial search ended in 2021). These studies were subsequently further screened to narrow the scope to waveform studies in medical education. *Stage 2 (updated screening): The second stage included the studies from the initial screening stage and the citation snowballing. Studies from the updated database searches of EEG studies from 2022 to 2024 were subsequently added. These studies were screened and yielded 142 studies for eligibility assessment, with 82 studies remaining after application of eligibility criteria. *Stage 3 (EEG instruction studies): 50 of the 82 studies were excluded as they represented EKG studies, and 1 additional EEG study was excluded as it did not meet eligibility criteria. After the exclusion of these 51 studies, 31 studies underwent data extraction and 2 additional studies were added for data extraction based on search of citations from the 2022–2024 updated database search from stage 2. The abovementioned multistage iterative process resulted in the final number of 33 studies included in this scoping review. CENTRAL = Cochrane Central Register of Controlled Trials; CINAHL = Cumulative Index to Nursing and Allied Health Literature; EKG = electrocardiography; ERIC = Education Resources Information Center; PRISMA = Preferred Reporting Items for Systematic Reviews and Meta-Analyses.

We conducted our scoping review following an enhanced framework,^7^ which builds on Arksey and O'Malley's original methodology.^8^ As a point of methodological clarification, a scoping review is a review type distinct from a systematic review in that it seeks to map and describe the breadth, nature, and gaps of existing literature on a topic through descriptive research questions with broad inclusion criteria to capture multiple evidence types and heterogeneous literature. Our approach included the following steps: identifying the research question; identifying relevant studies; study selection; charting the data; collating, summarizing, and reporting results. For the sixth stage, which is recommend as optional expert consultation, the authors (A.F., J.R., M..M., V.v.K.) fulfilled the role of content experts.

We report the findings in accordance with the Preferred Reporting Items for Systematic Reviews and Meta-Analyses extension for Scoping Reviews.^9^

### Review Questions

We derived the following research questions through iterative discussion within the study team:

1. What are educational theoretical frameworks used to underpin EEG instruction in medical education?
2. What methodologies are used in studies of EEG instruction in medical education?
3. Who are the learners in studies of EEG instruction in medical education?
4. What instructional methods and tools are used to teach EEG in medical education?
5. What educational assessments are described in studies of EEG instruction in medical education?
6. What are the gaps in the literature related to EEG instruction in medical education (i.e., areas to focus research)?

### Eligibility Criteria

The study team discussed initial inclusion and exclusion criteria and iteratively modified them to align with the review questions (Table 1).

**Table 1:** Inclusion and Exclusion Criteria

	Inclusion criteria	Exclusion criteria
Date range	No restrictions	
Literature type	No restrictions on literature type or publication source	Literature not in English or Spanish
Study methodology	No restrictions	
Setting	Medical education (undergraduate, graduate, or continuous medical education)	Nonmedical education setting
Participants/population	Learners in medical education (undergraduate, graduate, or continuous medical education)	Learners not in medical education
Methods and interventions	The article includes a teaching method or instructional tool for the purpose of EEG teaching (i.e., teaching related to EEG waveforms)	No instructional method or tool described
		Artificial intelligence tool described but not used for instruction
		Studies teaching other types of EEG (e.g., quantitative EEG)
Educational rationale and assessment	Defined educational rationale and assessment	No description of an educational rationale, assessment method, or outcome
	Rationale for learner assessment beyond evaluation of the program	Program evaluation without learner assessment

### Information Sources and Search Strategy

Our study team included an information specialist (G.L.) who developed a primary search strategy with the team (eAppendices 1 and 2). The initial strategy, focused on teaching visual diagnosis, was first developed for PubMed/MEDLINE (National Library of Medicine) and then translated with database-specific structured vocabulary in the following databases: Scopus, PsycINFO, Cumulative Index to Nursing and Allied Health Literature, CENTRAL, and Education Resources Information Center. We conducted these initial searches (stage 1: initial screening stage) on December 30, 2021, covering publications from the inception of the databases to the end of 2021 (eAppendix 1). Subsequently, we developed an updated search strategy (as part of stage 2: updated screening), specifically targeting EEG studies from 2022 to 2024 to complement the abovementioned initial search (eAppendix 2). This revised strategy was applied to the same databases as the initial search. These supplemental, updated searches were conducted on May 30, 2024, covering publications from the beginning of 2022 to the date of the search. Records were imported and managed in Covidence.^10^

### Selection of Sources of Evidence

The title and abstract screening of studies from the initial search in stage 1 was divided into 2 dyads (A.F./U.V. and J.B.R./A.P.), with each dyad screening half of the studies. The subsequent title and abstract screening based on the narrowed criteria in stage 1 was performed by 1 dyad (A.F./D.F.B.). The title and abstract screening for stage 2 was divided into 2 dyads (A.F./M.A. and A.F./D.F.B.). The full-text screening (study assessment for eligibility) for stage 2 was performed by 1 dyad (A.F./D.F.B.). Stage 3 assessment for eligibility was performed by A. Fernandez. Discrepancy in the screening decisions was resolved by a third reviewer (D.P.). We used the Preferred Reporting Items for Systematic Reviews and Meta-Analyses 2020 flow diagram to summarize the screening process (Figure).

### Data Charting Process and Data Items

We developed a data extraction form through an iterative process involving piloted data extraction and multiple team discussions and reviews. The final form was refined based on insights gained during these meetings, ensuring that the data extraction categories aligned with the review questions (Table 2). Data extraction was performed by A. Fernandez in Excel, with ongoing support and review from D.F. Balmer. This collaborative approach ensured consistency and accuracy in the data extraction process.

**Table 2:** Data Extraction Form

Data extraction categories	Coding variables
Author(s)	
Year of publication	
Publication source	Journal name
Study origin	Country (or countries) of study origin and institution(s)
Type of learners and no. of participants	For example,:medical students, residents, fellows
Theoretical frameworks or models	Presence (or absence) of an explicitly reported theoretical framework or model. If present, they were categorized into minimal, moderate, or major using the definition by Kumasi et al.^11^
Research methodologies	For example: quantitative, qualitative
Instructional approaches	For example: lecture, online modules, apprenticeship
Assessment approaches	For example: multiple-choice test, tests with EEG samples
Program evaluation	Presence of program evaluation of the educational intervention
Kirkpatrick levels of evaluation	Categorization by Kirkpatrick levels^12,13^

### Critical Appraisal of Individual Sources of Evidence and Synthesis of Results

Consistent with guidance on conducting scoping reviews, we did not perform a structured quality appraisal of identified studies.^7,8,14^ Quantitative data were summarized using descriptive statistics (including counts and percentages for the different categories when applicable). We worked collaboratively to synthesize a narrative account of our findings including the extent and range of categorized instruction and assessment approaches along with developing a visual display (Table 3).

**Table 3:** Visual Display of the Instructional, Assessment, and Program Evaluation Approaches for the 33 Studies in This Scoping Review

	Instructional approaches			Assessment approaches			
Year of publication	Traditional didactic instruction	Online-based instruction	Experiential learning	MCQ tests	Tests based on EEG samples	Standardized summative tests	Program evaluation surveys
1995	McCall				McCall		
2004		Leira			Leira		
2008	Fahy		Fahy	Fahy			
2009	Fahy		Fahy	Fahy			
2010	Chau			Chau			
2011		Bensalem	Bensalem	Bensalem			
2014	Chau		Chau	Chau			
	Fahy		Fahy	Fahy			
2015	Fahy	Fahy	Fahy	Fahy			
	Kobayashi				Kobayashi		
		Vasilopoulos	Vasilopoulos	Vasilopoulos			Vasilopoulos
2016			Venkatraman		Venkatraman		
		Weber		Weber	Weber		
2017		Moeller		Moeller			Moeller
2018			Dericioglu		Dericioglu		
2019		Chari		Chari	Chari		
	Fahy		Fahy			Fahy	
	Ding	Ding			Ding	Ding	
2020		Fahy	Fahy	Fahy	Fahy		Fahy
	Lybeck	Lybeck			Lybeck		
	Yadala			Yadala			Yadala
2021		Fahy			Fahy		
	Legriel			Legriel			
	Pan		Pan		Pan		
	Kyriakopoulos	Kyriakopoulos	Kyriakopoulos		Kyriakopoulos	Kyriakopoulos	Kyriakopoulos
2022		Asukile		Asukile	Asukile		Asukile
		Fahy	Fahy	Fahy	Fahy		Fahy
	Nascimento			Nascimento			
	Kural	Kural			Kural		
2023		Barfuss			Barfuss		
	Passiak	Passiak	Passiak	Passiak			
2024		Nascimento			Nascimento		
		Sheikh		Sheikh			Sheikh

Abbreviation: MCQ = multiple-choice question.

Each row represents 1 study.

The first author's last name for each study is indicated within the blocks.

The studies are arranged by year of publication, with the earliest studies at the top.

### Data Availability

Data not published within this article will be made available by request from any qualified investigator.

## Results

We identified 6,676 records from the initial search and 345 records from the updated 2022–2024 search; a total of 2,882 duplicated records were removed. The multistage screening process resulted in the extraction of 33 studies for inclusion in the scoping review.

### General Characteristics

The landscape of EEG instruction research has evolved significantly over the past 2 decades. With the exception of a single study from 1995, all publications emerged since 2004, with a notable surge in the past decade. Indeed, 82% (27/33)^15–41^ of studies were published since 2014.

Geographically, North America dominated the field, producing 79% (26/33) of the studies.^16–18,20–25,28,31–35,37–47^ Studies predominantly originated from single institutions (76%, 25/33),^18–26,28,30,33–35,37–47^ with over a third (36%, 12/33)^18,20–25,37,42–45^ describing iterations of a single EEG educational initiative. The publication landscape reflected the interdisciplinary nature of EEG education. While neurology/neuroscience journals published most of the studies (61%, 20/33),^15,16,19,22,24–29,32–36,39,41–43,47^ other journal types, such as anesthesia (n = 5), critical care (n = 2), general medical (n = 2), and emergency medicine (n = 1) journals (30%, 10/33),^17,18,21,23,30,37,40,44–46^ also made significant contributions. Notably, education journals published only a small fraction (9%, 3/33).^20,31,38^

Study populations varied, with focus on neurology trainees (residents or fellows) in 42% (14/33) of the studies,^19,22,27,28,31–36,38–41^ compared with 30% (10/33)^18,21,23–25,42–45,47^ targeting trainees in other specialties such as anesthesiology, neurosurgery, psychiatry, or critical care medicine. Some studies (15%, 5/33)^15,16,29,37,46^ incorporated mixed learner populations while a few focused exclusively on medical students (6%, 2/33)^20,26^ or non-neurology faculty (6%, 2/33).^17,30^ Few studies specifically included targeted pediatric EEG teaching (9%, 3/33),^26,35,36^ teaching EEG from rapid EEG devices (3%, 1/33),^28^ or teaching situated in nonacademic/resource-limited settings (3%, 1/33).^15^ The median number of study participants was 20 (interquartile range 11–33), suggesting a predominance of smaller scale studies.

The use of theoretical underpinning was rarely described, with only 4 studies (12%, 4/33)^16,22,31,32^ explicitly using theoretical frameworks or models. Of interest, these theory-driven studies were all recent, published between 2017 and 2024, and drew on diverse educational theories or models, including an educational model^48^ to inform the development of a video-based curriculum,^31^ retrieval practice for an online EEG learning module,^22^ game-based learning for an online EEG learning tool,^16^ and technology-enhanced learning and retrieval practice to inform the development of content for an EEG educational program pilot trial.^32^ Based on categories^11^that describe the continuum of “theory talk,” these 4 studies demonstrated “major theory talk,” applying theoretical frameworks to inform study design and/or data analysis.

Methodologically, the field showed strong preference for quantitative approaches, with all included studies using quantitative methodologies. Two studies stood out as randomized controlled trials.^17,32^ Rigorous qualitative methodologies were notably missing.

### Instructional Approaches

Our analysis revealed a diverse landscape of EEG instruction, which clustered around 3 categories: traditional didactic instruction (i.e., lectures or workshops), online-based instruction (i.e., online modules and interactive online platforms), and experiential learning (i.e., practical experiences in the clinical setting including apprenticeship-based learning). Several studies described educational initiatives that blended multiple modalities (e.g., combination of online modules complemented by experiential learning).

Traditional didactic instruction remained a cornerstone of EEG education, with more than half of the studies (55%, 18/33)^18,20,21,24,26–30,33–35,40,41,43–45,47^ incorporating this approach. Lectures were the most common format, featured in 42% (14/33) of the studies.^18,20,21,24,26,29,30,33–35,43–45,47^ Workshops, defined as synchronous interactive sessions focusing on discussion and critical thinking, were less frequent but still significant, appearing in 18% (6/33) of the studies.^27,28,30,35,40,41^ The digital evolution in education was evident in our findings, with 58% (19/33) of studies^15–17,20,22,23,25,27,28,30–32,35–37,39,41,42,46^ incorporating online-based instruction. These ranged from simple online modules to sophisticated interactive platforms. Notably, more than a third of the studies using online tools (37%, 7/19)^15,16,22,23,25,32,39^ emphasized interactivity. Experiential learning with practical, hands-on experience remained a key component of EEG education. Almost half of the studies (45%, 15/33)^18–21,23–25,28,34,35,37,38,42,44,45^ included experiential learning through practical experiences in clinical settings.

### Assessment Approaches

Our review revealed a diverse landscape of learner assessment approaches in EEG education, which clustered around 3 categories: multiple-choice question tests, tests based on EEG samples, and standardized summative tests. Several studies used multiple assessment tools. Program evaluations focused on the intervention as a unit of analysis, not individual participants.

More than half of the studies (58%, 19/33)^15,17,18,20,21,23,25,29,31,33,35–37,39,40,42–45^ included a multiple-choice question test, typically administered as a pre-post test intervention design. Tests based on EEG samples were equally prevalent, featured in 55% (18/33) of the studies.^15–17,19,22,23,25–28,30,32,34,38,39,41,46,47^ These assessments often required free-text responses, or categorization of EEG findings, with answers compared with expert references. Only a small fraction of the studies (9%, 3/33)^24,28,41^ used standardized summative tests. Two of these studies administered the American Clinical Neurophysiology Society Terminology Certification test,^28,41^ whereas one used EEG-related questions from the American Board of Anesthesiology in-training examination.^24^ Almost a quarter of the studies (21%, 7/33)^15,17,21,29,31,34,44^ assessed participants longitudinally, often with serial tests beyond the pre-post intervention design. In addition, approximately a quarter of the studies (24%, 8/33)^15,23,25,28,31,36,37,40^ included surveys for program evaluation.

Based on Kirkpatrick levels of evaluation,^12,13^ almost all studies focused on level 1 and/or 2, evaluating reactions and/or learning, respectively. The exceptions were 2 observational studies,^28,38^ which represented level 3, demonstrating behavior change in actual clinical practice: one study^38^ followed a single resident through a 1-month EEG rotation while another^28^ compared on-call neurology residents' EEG interpretation with that of expert electroencephalographers.

Table 3 provides a visual display of the instructional, assessment, and program evaluation approaches for each of the 33 studies, ordered by year of publication.

## Discussion

This scoping review reveals a rapidly evolving landscape of EEG instruction, with a notable surge in publications over the past decade indicating growing recognition of its importance. The review demonstrates a trend toward comprehensive, multifaceted approaches to EEG instruction, integrating traditional lectures with interactive online tools and hands-on clinical experiences. This integration may create potentially more effective learning environments, reflecting the complex nature of EEG education. Despite technological advancements, face-to-face instruction continues to play a crucial role, complemented by a shift toward more engaging and dynamic digital learning experiences. The high proportion of studies incorporating experiential learning underscores the enduring importance of apprenticeship-based learning and real-world application in developing EEG interpretation skills. These findings, taken together, not only reflect the current state of EEG instruction but also point toward actionable steps that educators and curriculum designers can take to enhance EEG instruction across diverse contexts.

Although assessment approaches primarily focused on knowledge attainment or retention, many studies used multiple tools to comprehensively evaluate learner progress. While multiple-choice question and EEG sample-based tests dominate, likely owing to their efficiency and ability to assess practical, applied knowledge, the emergence of longitudinal assessments and higher level assessments points to a growing sophistication in EEG education. Of note, the limited use of standardized tests suggests an opportunity for developing more widely accepted, standardized assessment tools in EEG education. There are additional opportunities for growth, particularly in terms of theoretical grounding, methodological diversity, and collaborative research efforts.

The findings from this scoping review can inform future research and educational interventions, pointing toward 3 areas for growth and improvement outlined further that could be explored in future EEG education studies.

**Increase the rigor of research that investigates the assessment of learning and evaluation of EEG educational interventions by applying relevant theories.** We found a paucity of theoretical frameworks in the studies in this scoping review. Theoretical grounding of future studies could help explain the underlying mechanisms of teaching and learning that facilitate the acquisition of EEG skills.^49^ Another area that could be expanded in future studies is the assessment of knowledge attainment or retention by exploring practice changes within workplace learning, as well as the nuances of EEG teaching and learning through qualitative methodologies.^50,51^ The current focus on evaluating learner reactions and specific EEG skills or EEG topics can also be further expanded to investigate the underlying mechanisms of learning. Studies in other visually rich fields, such as pathology and radiology, offer valuable examples to follow when examining the underlying mechanisms of visual processing and learning.^52,53^ Program evaluations can also complement the assessment of learner reactions and satisfaction, with a deeper exploration to help explain why interventions in those programs succeed or fail.^54,55^

Overall, we advocate for assessments that measure higher levels of learning, adoption of qualitative and mixed-methods methodologies, and incorporation of robust program evaluation to build a more comprehensive understanding of EEG teaching and learning. Faculty development programs could further support educators in selecting and applying relevant educational theories that align with their instructional goals.

**Advance the scope of research that investigates EEG instruction through collaboration across geographic regions and borrowing from related disciplines.** We found a restricted geographic and disciplinary scope of EEG instruction research because most studies were single-center, originated in academic settings in North America, and focused on adult EEG teaching, resulting in context-dependent study findings, limiting their transferability. In addition, because most studies were published in clinical journals, there is an opportunity for future EEG education studies to join a different scholarly conversation,^56^ for example, around the scholarship of visual diagnosis teaching and learning in education-based journals. Of note, the recent launch of the journal Neurology: Education offers a dedicated space for education literature in the field.

To enhance transferability and relevance, future EEG curricular studies could be co-developed across institutions in a collaborative model^57^ and tested in both academic and community-based settings. For example, partnerships between high-resource and low-resource environments could foster innovations in scalable online instruction or asynchronous feedback models. Overall, we suggest expanding the research context through multicenter studies and broadening geographic and disciplinary diversity to include nonacademic/resource-limited settings and pediatric EEG teaching.

In addition, we encourage scholarly expansion by incorporating theoretical learnings and practical instructional and assessment approaches from other visual fields. Educators might benefit from examining studies from these visual fields that share cognitive demands with EEG interpretation to inform their future work. Examples include studies from EKG education exploring cognitive load in the setting of worked examples^58^ or the challenges and nuances of interventions aimed at the diagnostic reasoning process.^59^ Other studies include exploring systematic viewing of chest X-rays^60^ or the intricate quantitative details of generating learning trajectories derived from learner classification of elbow radiograph and EKG data sets and their implications for adaptive learning.^61^

**Enhance the precision of EEG instruction by attending to the educational context and learner populations.** The studies in this scoping review describe a range of instructional and assessment approaches (Table 3 provides a visual display). A temporal change is seen with the availability of technological advancements in instructional and assessment tools; for instance, more interactive online tools have appeared in recent years. These technological advances will likely rapidly accelerate with the integration of artificial intelligence in EEG interpretation and its use in developing new instructional and assessment tools.

Because EEG has utility across multiple clinical environments, EEG education should seek alignment with the clinical specialty context and the ultimate applications of the acquired skill by the targeted learners. Although most studies in this review focus on neurology trainees, several target other learner populations (e.g., anesthesiology residents or critical care medicine fellows), pointing toward a perceived need to fill educational gaps and a need for EEG training beyond the field of neurology. A comprehensive discussion of EEG competency and formal certification in EEG interpretation^62^ is beyond this article. Nonetheless, the depth of familiarity with EEG interpretation and the applications of that interpretation can differ across different specialty contexts. Future studies could incorporate contextual questions when building their conceptual frameworks^63^ such as the following: In what clinical context(s) will the learner be interpreting EEG? Do they need to learn the basics to know when to order an EEG and understand what they read in an EEG report? Do they need to learn how to interpret an EEG and achieve mastery of the skill with the purpose of EEG interpretation in clinical practice? Tailoring instructional and assessment design^64,65^ for different learner populations may be a useful focus for future studies (e.g., studies could explore instructional approaches in the setting where the goal is to achieve mastery in EEG interpretation for clinical practice vs achieving a basic understanding of EEG principles). Educators could also inform their studies on the intended level of EEG competency (competency vs proficiency vs mastery) for their target learners at the outset. This clarity could facilitate alignment of instructional methods, assessment tools, and feedback strategies with learner needs. For instance, a flipped classroom model^66^ with annotated EEG cases may suffice for non-neurology learners, whereas neurology learners may benefit from longitudinal mentored interpretation of EEGs integrated into clinical rotations.^67,68^ Findings from such studies could also inform the allocation of available educational resources for any given context, appreciating that EEG training and training regulations differ in North America vs Europe and other parts of the world.

The 3 areas discussed above offer a substrate for practical suggestions that can be the basis for future research in EEG education, which are summarized in Table 4.

**Table 4:** Practical Recommendations for Advancing EEG Education

Support faculty development with appropriate theoretical grounding in EEG education
Conduct research on higher order learning processes to explore how and why EEG learning occurs
Incorporate qualitative and mixed-methods research to capture complex educational phenomena
Integrate program evaluation into EEG education initiatives to assess design and implementation
Promote multi-institutional collaborations to enhance transferability and relevance across varied settings
Expand EEG education research to underrepresented contexts, including diverse institutional or resource settings
Adapt and study instructional approaches from other visual diagnostic fields (e.g., EKG, radiology, and pathology)
Explore the integration of artificial intelligence in instructional and assessment strategies for EEG education
Define EEG competencies aligned with specific learner audiences

Abbreviation EKG = electrocardiography.

We acknowledge the limitations of this scoping review. For example, the search strategy only included indexed journals. Newer journals that were not yet indexed at the time of the searches such as Neurology: Education, which has articles about EEG education, are not included in this scoping review. The wide scope of the initial search terms prolonged the process of focusing on EEG instruction studies. The inconsistent use of terms (e.g., theory or evaluation) in the studies required interpretation during the extraction process. The searches could also have missed relevant studies that lacked indexing terms used in the search strategy. In addition, we did not include studies of quantitative EEG teaching or learner populations such as EEG technologists, and there was a paucity of EEG teaching from rapid EEG devices. These areas could be the focus of future studies. Finally, medical education and EEG instruction will continue to advance with technological breakthroughs, especially the implementation of artificial intelligence.^69^ Artificial intelligence will be an important area to integrate into the educational principles and approaches described in this scoping review.

This scoping review makes an important contribution to the literature by providing a landscape of research on EEG instruction and calling out areas for growth and improvement that could support the articulation of meaningful questions, theoretical frameworks, and study designs for future research. The findings from this review can serve as a guide for the development of future studies, including multicenter collaborative studies on EEG curriculum development. This scoping review can further inform the research questions and gaps to be addressed in these studies.

## Glossary

EKG: electrocardiography
